# Disentangling personalized treatment effects from “time-of-the-day” confounding in mobile health studies

**DOI:** 10.1371/journal.pone.0271766

**Published:** 2022-08-04

**Authors:** Elias Chaibub Neto, Thanneer M. Perumal, Abhishek Pratap, Aryton Tediarjo, Brian M. Bot, Lara Mangravite, Larsson Omberg

**Affiliations:** Sage Bionetworks, Seattle, Washington, United States of America; Public Library of Science, UNITED STATES

## Abstract

Ideally, a patient’s response to medication can be monitored by measuring changes in performance of some activity. In observational studies, however, any detected association between treatment (“on-medication” vs “off-medication”) and the outcome (performance in the activity) might be due to confounders. In particular, causal inferences at the personalized level are especially vulnerable to confounding effects that arise in a cyclic fashion. For quick acting medications, effects can be confounded by circadian rhythms and daily routines. Using the time-of-the-day as a surrogate for these confounders and the performance measurements as captured on a smartphone, we propose a personalized statistical approach to disentangle putative treatment and “time-of-the-day” effects, that leverages conditional independence relations spanned by causal graphical models involving the treatment, time-of-the-day, and outcome variables. Our approach is based on conditional independence tests implemented via standard and temporal linear regression models. Using synthetic data, we investigate when and how residual autocorrelation can affect the standard tests, and how time series modeling (namely, ARIMA and robust regression via HAC covariance matrix estimators) can remedy these issues. In particular, our simulations illustrate that when patients perform their activities in a paired fashion, positive autocorrelation can lead to conservative results for the standard regression approach (i.e., lead to deflated true positive detection), whereas negative autocorrelation can lead to anticonservative behavior (i.e., lead to inflated false positive detection). The adoption of time series methods, on the other hand, leads to well controlled type I error rates. We illustrate the application of our methodology with data from a Parkinson’s disease mobile health study.

## 1 Introduction

Smartphones offer a unique opportunity to develop large scale studies of human health [1–3]. Features extracted from data collected by accelerometers, microphones, and touch screen sensors can provide objective measurements of human health and disease [4]. In particular, smartphones have been used in diagnostic applications [5, 6], as well as, to monitor if a patient is likely responding to its medication [7, 8].

Here, we show how to disentangle personalized treatment and “time-of-the-day” effects in observational mobile health studies (an earlier version of the methodology described in this paper, together with some additional methodology for the assessment of identity confounding in mobile health studies [9], is available on arXiv [10]). The present work was motivated by the analysis of mobile heath data collected during the first 6 months of the mPower (mobile Parkinson’s observatory for worldwide evidence-based research) study [11]. In this purely observational study, each Parkinson’s disease participant is asked to perform activity tasks [11], both before and after the participant has taken dopaminergic medication. Raw sensor data collected from each task is processed into a number of distinct activity specific features (which are used to measure the participant’s performance in the activity). Because the activities are performed by the patient on a daily basis, over a long period of time, the processed data corresponds to a time series of feature measurements, annotated according to whether the measurement was taken before or after the participant has taken medication.

**Fig 1:**
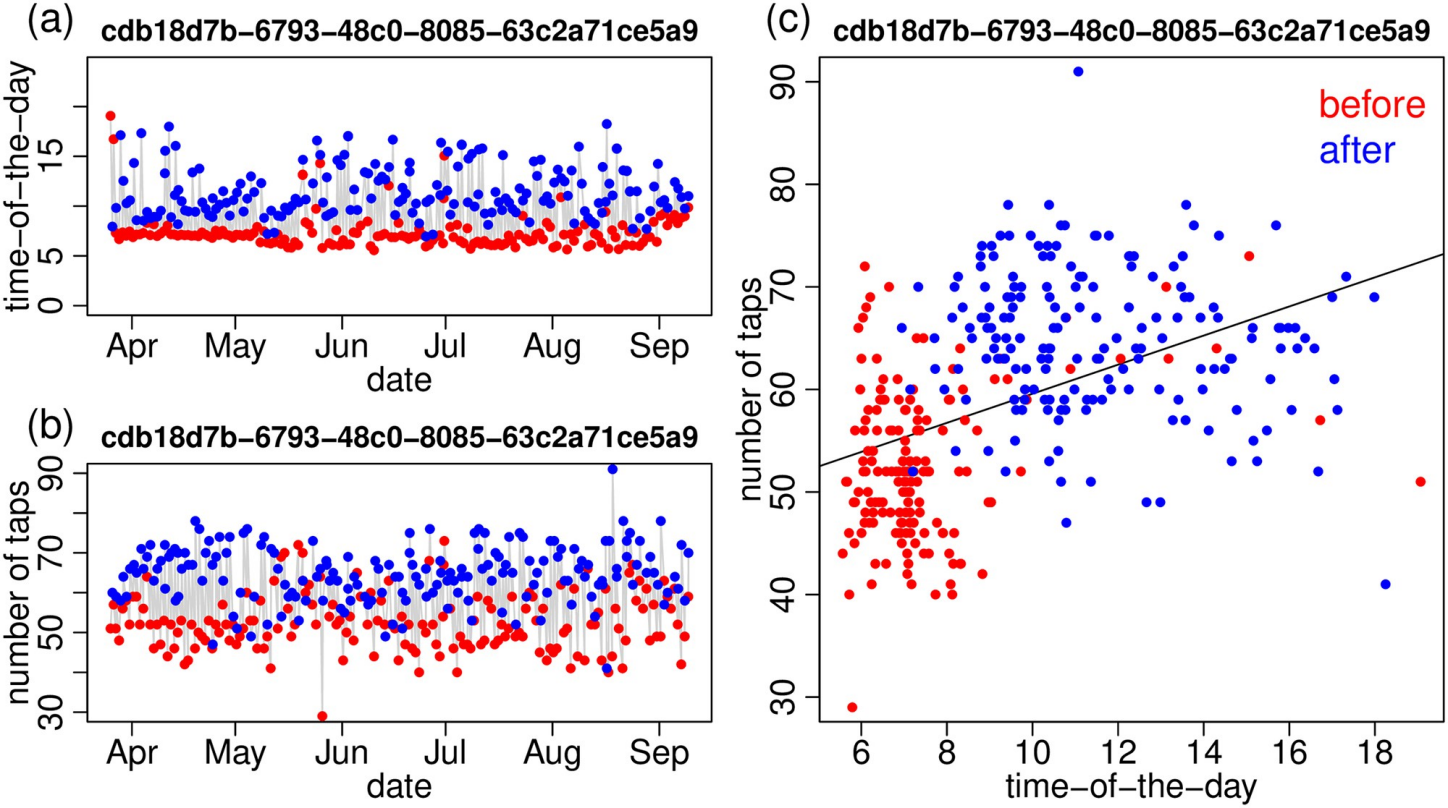
Marginal associations between treatment (before/after medication status), time-of-the-day and number of taps, for one study participant. Panel a shows that the participant usually performs the before medication tapping tasks (red dots) earlier in the day than the after medication tasks (blue dots). Panel b shows the participant also tends to achieve better performance (larger number of taps) in tasks performed after medication. Panel c, nonetheless, also shows that large number of taps tends to be associated with later times. Hence, it is possible that the medication and/or circadian rhythms/daily routine activities might be responsible for the difference in performance between the before and after medication tapping tasks observed in this participant.

As the data consists of long time-series for each participant we are able to focus on personalized analyses where we can observe individualized response to medication. Different from traditional trial designs, where the goal is to establish treatment efficacy at a population level for a target cohort of patients [12, 13], our goal is to determine whether a particular patient is responding to medication (as measured by the difference in the participant’s performance when medicated in comparison to when the participant is unmedicated). However, since mPower is an observational study, the associations observed between treatment and outcome measurements might be due to unmeasured confounders, and it is not possible to conclude with certainty that a difference in performance is actually due to the medication. In particular, causal inferences at the personalized level are especially vulnerable to confounding effects that arise in a cyclic fashion over the day (such as circadian rhythms and daily routine activities). For instance, we observed in the data (Fig 1a) that some participants tended to perform the “before medication” activities earlier in the day than the “after medication” activities. For these participants, it is not possible to conclude that an observed improvement in performance between activities performed before versus after medication are suggestive of a medication effect, since the difference in performance might be due to daily cyclic confounders (Fig 1b and 1c).

Fortunately, the time-of-the-day that the activity is performed is usually recorded by mobile health apps, and we can use it as a surrogate variable for confounding caused by circadian rhythms and daily routine in our analyses. Arguably, these sources of short-term cyclic confounding account for the bulk of confounding issues in personalized analyses. We clarify, nonetheless, that in observational studies we can never guarantee that the inferences are completely free of unmeasured confounding biases. For this reason, throughout the paper we refer to causal effects as “putative causal effects” to reinforce the point that, although unlikely, these “effects” might still correspond to spurious correlations generated by longer-term sources of confounding that are not captured by the time-of-the-day variable. (The reason why these longer-term sources of confounding are much less likely to affect our results is because they require a peculiar synchronization of events. The following hypothetical example clarifies this point. For instance, consider a participant that: (i) always drinks alcohol throughout the day over the weekends, but never drinks during weekdays; (ii) do not take medication over the weekends; (iii) always performs the activities off-medication during the weekends; and (iv) usually performs activities on-medication during the weekdays. In this scenario, alcohol consumption can be a confounder, since it is associated with taking medication, and also with performance on the activities, as we would expect worse performance under the influence of alcohol, than when sober. Hence, for this participant, it is possible that sobriety rather than medication is driving the better performance over the weekdays. Our point, however, is that while scenarios such as this one are not impossible, they require these very special synchronization of events, which make then much less likely in comparison to daily routines and circadian rhythms. Still, because we acknowledge the possibility that longer-term unmeasured confounders might still bias our results, throughout the text we qualify the use of the term “effect” with the adjective “putative” to acknowledge the possibility that our detected “effects” might still correspond to spurious correlations generated by unmeasured longer-term confounding).

Essentially, our goal is to learn the causal relations between the treatment, time-of-the-day and outcome variables from the data. To this end, we employ causal graphical models [14] represented by directed acyclic graphs (DAGs) involving these 3 variables. Generally speaking, there are 25 distinct DAGs containing 3 nodes. However, we can a priori discard DAG structures where the output variable have a causal influence on the treatment and/or time-of-the-day variables (since in our application it is reasonable to expect that the time-of-the-day that the activity was performed, or whether the participant was on- or off-medication can influence the participant’s performance on an activity, but not the other way around). On the other hand, because we cannot a priori specify the causal direction between the treatment and the time-of-the-day variables, we are still left with 9 distinct causal models. But, most importantly, as fully described in the next section, the conditional independence relations spanned by these 9 models allows us to disentangle treatment and time-of-the-day effects, irrespective of the causal relation between the treatment and time-of-the-day variables. (To see why we cannot specify a priori the causal direction between the treatment and the time-of-the-day variables, note that if at a given day the participant decided to perform the activity task in the afternoon, and he/she usually takes the medication at lunch time, we have that the participant’s decision about doing the task in the afternoon caused the treatment to be “medicated”. Conversely, if the participant first decided that he/she would do the task after taking medication, than we have the situation where the treatment determined that activity was done in the afternoon).

Mechanistically, our approach is based on conditional independence tests implemented via temporal and standard regression models, and represents an improvement over a previous approach in the literature [7] where the longitudinal aspect of the data is ignored. Using synthetic data, we discuss when and how residual autocorrelation can inflate (or deflate) the p-values from standard regression models when the time series structure of the data is ignored, and propose the use of ARIMA processes [15] and heteroscedastic and autocorrelation consistent estimators of covariance [16] as remedies to these issues. We illustrate the application of the proposed methods to a subset of the tapping activity data collected during the first six months of the mPower study [11].

While in this paper we illustrate the application of our proposed method to disentangle putative medication effects from time-of-the-day effects in the mPower data, the methodology is more general and can be applied to other mobile health studies that aim to disentangle rapid-acting treatment effects from time-of-the-day effects. By rapid-acting treatments we mean any interventions that have an immediate/quick effect on the subject that receives the intervention. Such interventions include not only rapid-acting pharmacological interventions (such as the dopaminergic medications for Parkinson’s patients), but also behavioral interventions aiming at, for example, managing depression, pain, or sleep using text messaging. For instance, in the context of depression symptoms, consider the inclusion of messaging interventions in a study such as BiAffect [17], where depression is monitored by keyboard dynamics which are highly affected by diurnal variations [17, 18]. (For example, suppose that participants in a study such as Biaffect were to receive multiple text messaging interventions with distinct tips/strategies for managing their depression, according to a randomized schedule. Suppose that the goal is to investigate the effectiveness of the different tips/strategies, and that the degree of depression is assessed passively by measuring, for example, key-stroke dynamic features such as the number/length of text messages sent by the participants in a fixed interval after receiving their depression messaging intervention. Because time-of-the-day is known to influence key-stroke dynamics [17, 18] it will likely be a potential confounder of this depression management intervention. Such a behavioral intervention study would also be a candidate for the application of our methodology).

The key requirements for the applicability of our proposed methodology is that the study records the outcome variables under the different treatment interventions (e.g., the number of taps on- and off-medication in our Parkinson’s disease study), as well as, the time-of-the-day when the rapid-acting intervention happened.

The rest of this paper is organized as follows. Section 2 describes the proposed statistical method, and is organized into the following 4 subsections. Section 2.1 describes how to disentangle putative treatment and time-of-the-day effects using conditional independence relations implied by simple causal diagrams involving only the treatment, time-of-the-day, and outcome variables. (It does not deal with complications arising from serial associations in the data, and is simply meant as an gentle introduction to causal discovery based on conditional independence tests.) Section 2.2 frames the same problem in the context of time series data, and proposes concrete conditional independence tests implemented via t-tests from standard and time series regression models. Section 2.3 provides a description and detailed illustrations (based on synthetic data) of the conditions under which autocorrelation may or may not impact the validity of standard t-tests. This subsection also illustrates how the proposed time series regression approaches can account for the serial association in the data and produce valid statistical inferences. Section 2.4 describes how to aggregate evidence across multiple outcome variables (i.e., multiple features) into a single statistical test for detecting putative treatment and/or putative time-of-the-day effects. Finally, Section 3 illustrates the application of our tests to data collected from an observational mobile health study in Parkinson’s disease, while Section 4 provides final remarks.

## 2 The statistical method

Throughout this paper, we let *X*, *T*, and *Y* represent, respectively, the treatment (i.e., “participant is medicated” vs “participant is unmedicated”), the time-of-the-day that the activity was done, and the performance on the activity task (i.e., the outcome variable, represented by an extracted feature from the tapping activity such as, for example, the number of taps). In the following we describe how we can use a subset of the conditional independence relationships spanned by the {*X*, *T*, *Y*} variables in order the determine whether a difference in performance might be due to a putative treatment or putative “time-of-the-day” effect (or still both).

### 2.1 Disentangling putative treatment effects from putative “time-of-the-day” effects

Under the assumption that *X*, *T*, and *Y* are not influenced by unmeasured confounders (as well as, assuming that the standard Markov property for directed acyclic graphs [19] and the faithfulness of the probability distribution to the graph structure [14] holds in the data), it is possible to use a subset of the conditional independence relationships spanned by the {*X*, *T*, *Y*} variables to determine if *X* has a causal effect on *Y*, or if *T* has a causal effect on *Y*, or if both *X* and *T* have causal effects on *Y*, irrespective of the causal relationship between *X* and *T*.

**Fig 2:**
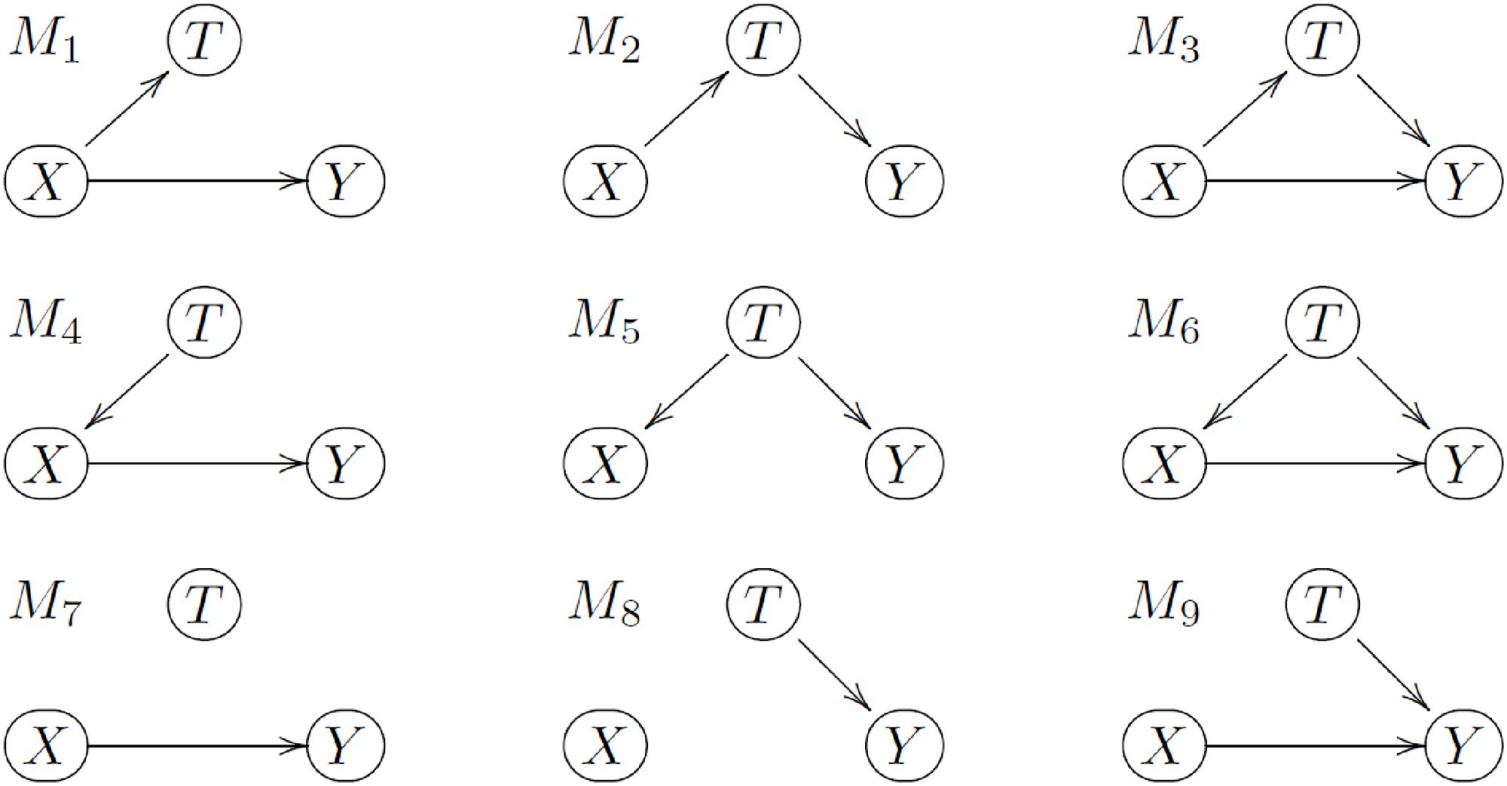
Putative causal models involving the *X*, *T*, and *Y* variables. No causal links from *Y* to *X* or *T* are not allowed.

Explicitly, consider the putative causal models listed in Fig 2.

Models *M*_1_ and *M*_4_ are indistinguishable in terms of conditional independence relationships. In the language of graphical models [19], *M*_1_ and *M*_4_ are Markov equivalent. (A simple graphical criterion for determining if two directed and acyclic graphs (DAGs) are Marvov equivalent is to inspect if the DAGs have the same skeleton and the same set of v-structures [20], where the skeleton of a DAG is obtained by replacing the directed edges by undirect ones, and a v-structure is composed by two converging arrows whose tails are not connected by an arrow. For instance, models *M*_1_ and *M*_4_ have the same skeleton, *T*—*X*—*Y*, and the same set of v-structures, namely, no v-structures, and we say that models *M*_1_ and *M*_4_ belong to the same equivalence class.) Note that while *M*_1_ and *M*_4_ differ with respect to the causal relation between *X* and *T* (where *X* → *T* in *M*_1_, and *X* ← *T* in *M*_4_), both models represent a causal effect of the treatment on the outcome (i.e., *X* → *Y*). Similarly, *M*_2_ and *M*_5_ are Markov equivalent and depict an effect of the time-of-the-day on the outcome, but no treatment effect, while *M*_3_ and *M*_6_ are Markov equivalent and represent the case where both treatment and time-of-the-day effects influence the outcome. Models *M*_7_, *M*_8_, and *M*_9_ represent, respectively, the causal DAGs for treatment, time-of-the-day, and both effects in the case where *X* and *T* are not even associated.

The subset of the conditional independence relations that can be used to distinguish between the 6 equivalence classes of models, {*M*_1_, *M*_4_}, {*M*_2_, *M*_5_}, {*M*_3_, *M*_6_}, *M*_7_, *M*_8_, and *M*_9_, is given in Table 1, where we adopt the notation ⫫ and ⫫ to describe statistical independence and dependence, respectively (and *A* ⫫ *B*∣*C*, to describe that *A* is independent of *B* conditional on *C*).

**Table 1:** Subset of the conditional independence relations, spanned by the causal models in Fig 2, that are sufficient to distinguish between the 6 equivalence classes of models: {*M*_1_, *M*_4_}, {*M*_2_, *M*_5_}, {*M*_3_, *M*_6_}, *M*_7_, *M*_8_, and *M*_9_.

Models	*T*, *X*	*Y*, *X*	*Y*, *T*	*Y*, *X* ∣ *T*	*Y*, *T* ∣ *X*	Putative effect
{*M*_1_, *M*_4_}	T⫫X	Y⫫X	Y⫫T	Y⫫X|T	*Y* ⫫ *T* ∣ *X*	treatment
{*M*_2_, *M*_5_}	T⫫X	Y⫫X	Y⫫T	*Y* ⫫ *X* ∣ *T*	Y⫫T|X	time of the day
{*M*_3_, *M*_6_}	T⫫X	Y⫫X	Y⫫T	Y⫫X|T	Y⫫T|X	both
*M*_7_	*T* ⫫ *X*	Y⫫X	*Y* ⫫ *T*	Y⫫X|T	*Y* ⫫ *T* ∣ *X*	treatment
*M*_8_	*T* ⫫ *X*	*Y* ⫫ *X*	Y⫫T	*Y* ⫫ *X* ∣ *T*	Y⫫T|X	time of the day
*M*_9_	*T* ⫫ *X*	Y⫫X	Y⫫T	Y⫫X|T	Y⫫T|X	both

By inspecting the results of the following 5 statistical tests,

H01:T⫫XvsH11:T⫫X,H02:Y⫫XvsH12:Y⫫X,H03:Y⫫TvsH13:Y⫫T,H04:Y⫫X∣TvsH14:Y⫫X∣T,H05:Y⫫T∣XvsH15:Y⫫T∣X,} (1)

we are able to determine which among (the equivalence classes of) models {*M*_1_, *M*_4_}, {*M*_2_, *M*_5_}, {*M*_3_, *M*_6_}, *M*_7_, *M*_8_, and *M*_9_, are supported by the data. For instance, the rejection of $H_{0}^{1}$, $H_{0}^{2}$, $H_{0}^{3}$, and $H_{0}^{4}$ together with the acceptance of $H_{0}^{5}$, indicates that the data supports *M*_1_ or *M*_4_, and that the association observed between *X* and *Y* might be due to a treatment effect. Similarly, the rejection of the $H_{0}^{3}$ and $H_{0}^{5}$ together with the acceptance of $H_{0}^{1}$, $H_{0}^{2}$, and $H_{0}^{4}$ indicates that the data supports *M*_8_.

**Fig 3:**
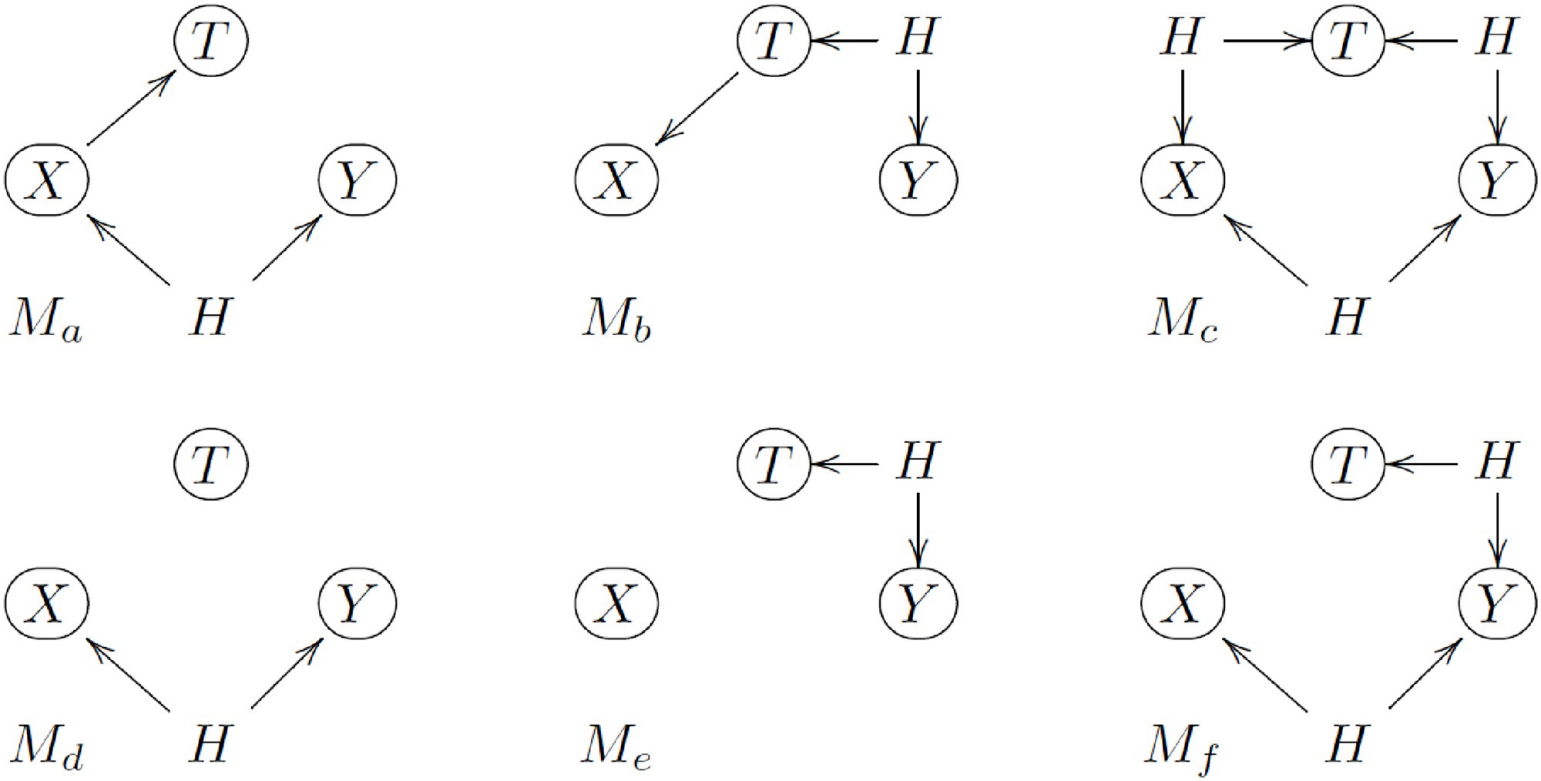
Alternative models involving unmeasured confounders, *H*.

It is important, nonetheless, to keep in mind that the results of the conditional independence tests are only consistent with the causal models in Fig 2 under the assumption that there are no unmeasured confounders. Hence, the proposed approach can only detect putative treatment and time-of-the-day effects. It is possible that, in reality, there are no treatment or time-of-the-day effects and the associations between the {*X*, *T*, *Y*} measurements are actually generated by confounding. For instance, the results of the conditional independence tests consistent with {*M*_1_, *M*_4_} are also consistent with *M*_*a*_ in Fig 3, where *H* represents an unmeasured confounder (other than short term cyclic confounders, such as circadian rhythms and daily routine schedules, for which the recorded time-of-the-day works as a surrogate variable). Similarly, test results consistent with models {*M*_2_, *M*_5_}, {*M*_3_, *M*_6_}, *M*_7_, *M*_8_, and *M*_9_, are also consistent with *M*_*b*_, *M*_*c*_, *M*_*d*_, *M*_*e*_, and *M*_*f*_, respectively.

Hence, while short term cyclic confounding usually accounts for the bulk of confounding issues in personalized analysis, in observational studies we can never guarantee that the estimated effects are free of unmeasured confounding biases, and any causal inferences will always require assumptions.

### 2.2 Accounting for serial association in the data

**Fig 4:**
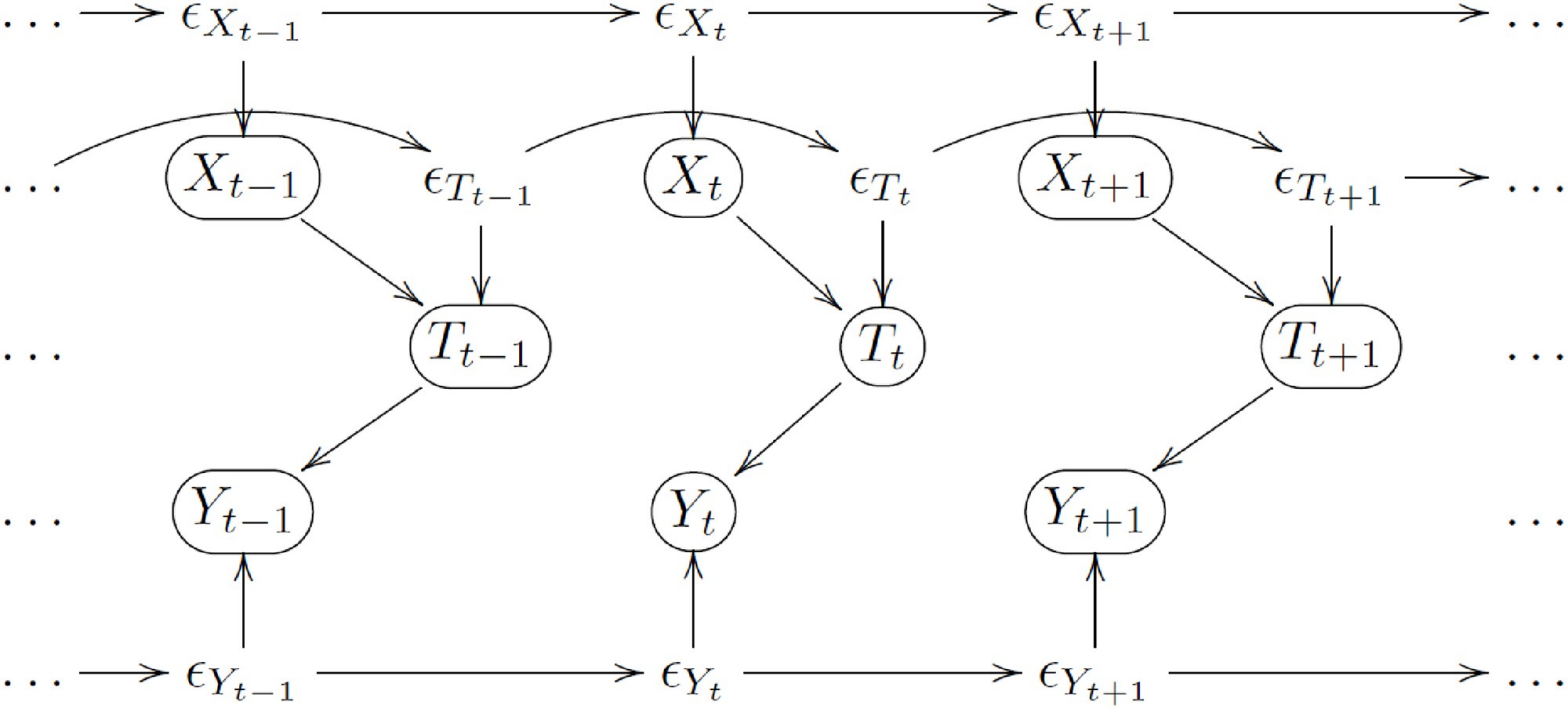
Dynamic version of model *M*_2_ in Fig 2.

In practice, we are interested in evaluating medication response at the personalized level and we analyze the longitudinal data from each participant separately. In this setting, the data from each participant corresponds to time series of treatment, time-of-the-day, and outcome variables, and it is natural to expect a serial correlation structure in the data. Therefore, the causal graphs that we are actually comparing are slightly more complicated than the graphs shown in Fig 2. For instance, Fig 4 represents a dynamic version of the causal graph *M*_2_ in Fig 2, where we assume that the serial correlation structure (that arises from the fact the the data comes from the same participant) is represented by the autoregressive structure of the residual error terms, $\in_{Y_{t}}$, $\in_{T_{t}}$, and $\in_{X_{t}}$. (Note that the model depicts a simple autoregressive serial association of order 1 only for illustrative purposes. In practice, the residual correlation structure is unknown and can be much more complicated).

For the dynamic version of model *M*_2_, the serially associated residual terms $\in_{Y_{t}}$ capture all factors that are not accounted for by the treatment and time-of-the-day variables, but that still influence the outcome variable over time. For instance, the performance of the participant at time *t* (measured by the *Y*_*t*_) will also depend on the participant’s current underlying physiological state, which is an unmeasured variable captured in the residual error term $\in_{Y_{t}}$. Since the participant’s physiological state should not change drastically over a short period of time (unless, of course, a major health disruption event happens), it is reasonable to expect that the participant’s physiological states (and, therefore, the residual error terms) will be autocorrelated over time. In a similar vein, the residual terms $\in_{T_{t}}$ capture all factors that are not accounted by the treatment, but that still influence the time-of-the-day variable over time, whereas the error terms $\in_{X_{t}}$ capture all factors that influence the treatment variable over time.

Most importantly, observe that the same 5 conditional independence tests can still be used to distinguish between the dynamic versions of models *M*_1_ to *M*_9_. For instance, it is still true that, at any time point *t*, the set of conditional independence relations associated with the dynamic version of model *M*_2_ are still given by Tt⫫Xt, Yt⫫Xt, Yt⫫Tt, *Y*_*t*_ ⫫ *X*_*t*_ | *X*_*t*_, and Yt⫫Tt|Xt (which correspond to the same conditional independence relations implied by the static version of *M*_2_). Observe, nonetheless, that the use of the longitudinal data to estimate these associations can only be justified under the assumptions that the causal effects between the variables are constant over time, and that the time series is stationary. (In time series analysis, the concept of stationarity captures the notion of regularity over time in the probabilistic behavior of the series [21]. A strictly stationary time series is defined as one for which the probabilistic behavior of every collection of variables, {*Y*_1_, *Y*_2_, …, *Y*_*k*_}, is identical to the shifted collection, {*Y*_1 + *j*_, *Y*_2 + *j*_, …, *Y*_*k* + *j*_}, for all *k* = 1, 2, …, all time points 1, …, *k*, and all shifts *j* = 0, ±1, ±2, …. The stationarity assumption plays a critical role in the analysis of time series data, since we do not typically have an independent and identically distributed sample, $\left\{Y_{t,1},Y_{t,2},\ldots ,Y_{t,n_{t}}\right\}$, of the variable *Y*_*t*_, but rather a single observation at each data point *Y*_*t*_. In this situation, with a single realization per time point, the assumption of stationarity allows us to compute standard sample statistics using the time series data [21]).

In our analyses, we adopt 3 distinct regression based approaches (which account for the serial correlation structures for the residuals in different ways). The first is a simple linear regression approach where we use standard t-tests for carrying out the 5 conditional independence tests in (1) based on 4 linear regression model fits,

$$\begin{array}{cc} T\; & =\;\mu \;+\;\beta_{T,X}\;X\;+\;\epsilon_{T}\;, \end{array}$$ (2)

$$\begin{array}{cc} Y\; & =\;\mu \;+\;\beta_{Y,X}\;X\;+\;\epsilon_{Y}\;, \end{array}$$ (3)

$$\begin{array}{cc} Y\; & =\;\mu \;+\;\beta_{Y,T}\;T\;+\;\epsilon_{Y}\;, \end{array}$$ (4)

$$\begin{array}{cc} Y\; & =\;\mu \;+\;\beta_{Y,X|T}\;X\;+\;\beta_{Y,T|X}\;T\;+\;\epsilon_{Y}\;, \end{array}$$ (5)

where the conditional independence tests in (1) are performed by testing,

$$\begin{array}{c} \begin{array}{ccc} H_{0}^{1}:\;\;\beta_{T,X}=0 & \text{vs} & H_{1}^{1}:\;\;\beta_{T,X}\neq 0,\\ H_{0}^{2}:\;\;\beta_{Y,X}=0 & \text{vs} & H_{1}^{2}:\;\;\beta_{Y,X}\neq 0,\\ H_{0}^{3}:\;\;\beta_{Y,T}=0 & \text{vs} & H_{1}^{3}:\;\;\beta_{Y,T}\neq 0,\\ H_{0}^{4}:\;\;\beta_{Y,X|T}=0 & \text{vs} & H_{1}^{4}:\;\;\beta_{Y,X|T}\neq 0,\\ H_{0}^{5}:\;\;\beta_{Y,T|X}=0 & \text{vs} & H_{1}^{5}:\;\;\beta_{Y,T|X}\neq 0. \end{array}\} \end{array}$$ (6)

We employ the lm function of the R software [22] base distribution for these analyses. Note that this approach naively assumes that the residuals of the linear regression fits are uncorrelated. Whether the serial association structure of the residuals impact the t-tests depends on whether the study participant performs the unmedicated and medicated activity tasks in a paired or un-paired (and random) fashion over time. We describe this point in more detail in the next section.

The second approach is based on regression with ARIMA errors modeling, where we basically fit the same 4 regression models in equations (2) to (5), but where the serial association of the residual errors are modeled according to an ARIMA (autoregressive integrated moving average) [15] process. Because the residual correlation structure is unknown, we employ the auto.arima function of the forecast R package [23] in order to first select the autoregressive, moving average, and differencing orders of the models that are used to test the hypothesis in (6).

The third approach is based on robust regression modeling with heteroscedasticity, and autocorrelation consistent (HAC) covariance matrix estimation. Non-parametric and kernel based HAC estimators are able to account for heteroscedasticity, and autocorrelation of unknown form, and can be used to construct statistical tests that are robust to violations of homoscedasticity and independent error assumptions. Here, we adopted the Newey-West HAC estimator [16], using Bartlett kernel, and the automatic bandwidth selection procedure described in reference [24], and implemented in the sandwich R package [25], in order to construct robust t-tests for the same 4 regression models in equations (2) to (5).

Finally, note that the regression fits in equations (2) to (5) should not be interpreted as linear structural causal models describing the causal relations between the variables. (For instance, the linear regression model in equation (2) is simply used to test for association between *T* and *X*, even though it might be the case that *T* has a causal effect on *X*.) In reality, these regression model fits are just a convenient way to perform conditional independence tests that can robustly account for heteroscedasticity and autocorrelation of unknown form in the data (when adopting HAC estimates), or can incorporate flexible serial association structures automatically learned from the data (when adopting regression with ARIMA errors). Observe, as well, that we are not really interested in estimating causal effects. The actual goal is to select the causal graph, among the 9 causal models in Fig 2, based on the observed conditional independencies in the data. Hence, our approach is closer in spirit to causal discovery algorithms (such as the PC algorithm [14]), but in a situation where we have partial domain knowledge which prohibits the causal links *Y* → *T* and *Y* → *X*, and where we are not interested in determining whether *X* → *T* or *T* → *X*.

### 2.3 On the validity of t-tests in the presence of serial correlation

Whether residual autocorrelation impacts the type I error rates of t-tests depends on whether a participant performs the activity tasks in a paired or unpaired (and close to random) fashion. For instance, if a participant tends to perform both the unmedicated and medicated activity tasks every day (so that the data is paired by day), the residual autocorrelation can have a strong impact on the t-test p-values. In the context of paired time series, it has been shown that in the presence of positive serial correlation the F-test distribution (and, hence, the equivalent t-test in our binary treatment case) has a thicker upper tail than when the serial correlation is zero, while for negative serial correlation the upper tail is thinner [26]. As a consequence, the t-tests are conservative in the presence of positive autocorrelation (i.e., the p-values tend to be larger than they should), and anti-conservative in the presence of negative autocorrelation (i.e., the p-values tend to be smaller than they should). On the other hand, if a participant tends to perform the tasks in an unpaired fashion (i.e., the before and an after medication tasks are not performed on the same day), with no particular structure about the order of the before/after tasks, then the presence of residual autocorrelation does not impact the p-value of a t-test, since the group labels (before/after medication) are exchangeable under the null hypothesis of no putative medication effect. (For further details see reference [7], where it was implicitly assumed that most participants performed the before/after medication tasks in an unpaired and mostly random fashion).

**Fig 5:**
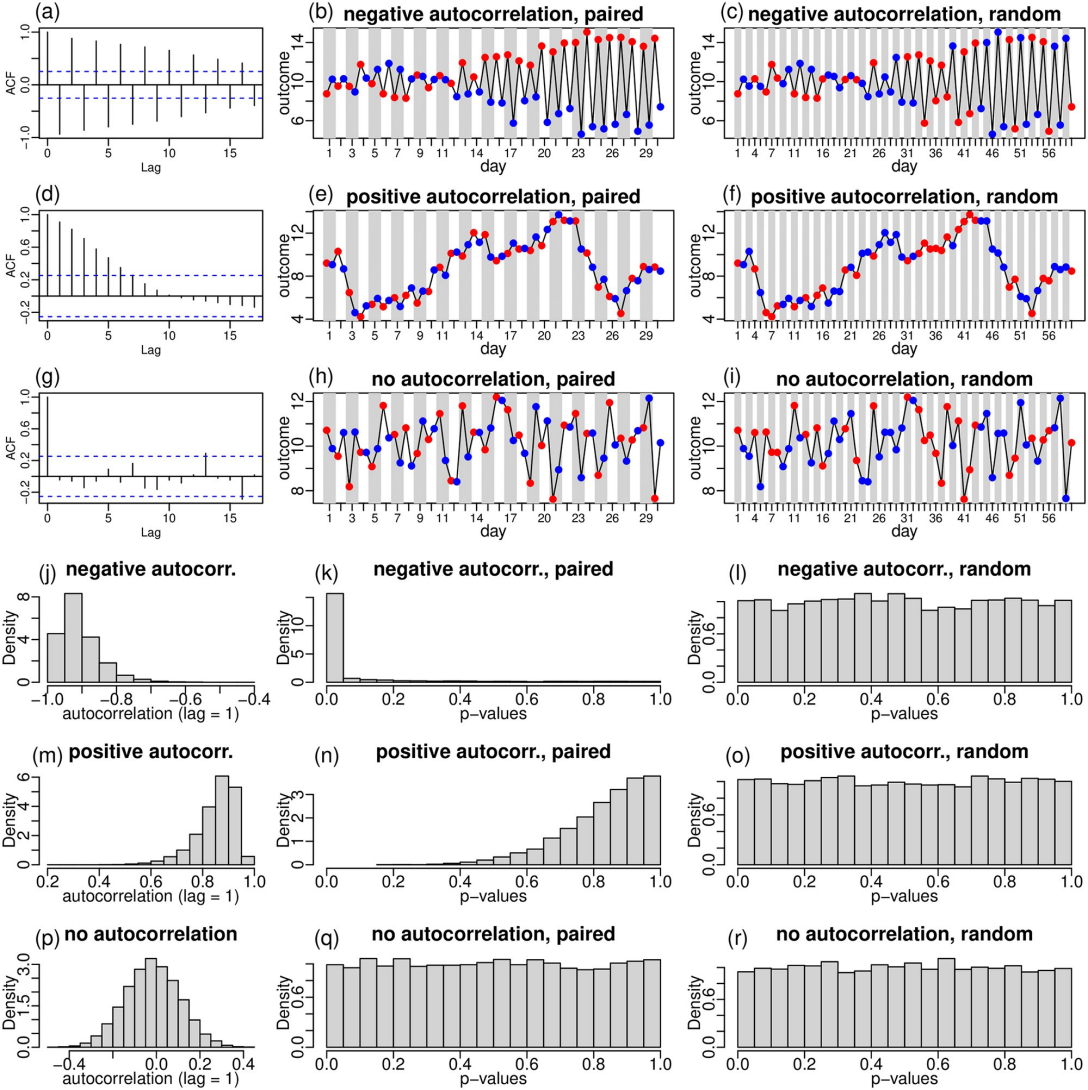
The effect of autocorrelation on t-tests. Here, we illustrate the effect of autocorrelation on t-tests using synthetic data simulated under the null hypothesis of no medication (or time-of-the-day effects). Panels a-c illustrate the negative autocorrelation case. Panel a shows the autocorrelation plot, of the outcome variable time series shown on panels b and c. Panel b show the time series for the outcome variable in the paired case. Panel c shows the same time-series on the random case. Red and blue dots correspond to activities performed “before” and “after” the participant has taken medication. Panels d-f illustrate the positive autocorrelation case, while panels g-i illustrate the no autocorrelation case. Panels j-l, m-o, and p-r, show the distributions of the empirical autocorrelation (lag = 1) estimates, and of t-test p-values, from 10,000 replications of the negative, positive, and no autocorrelation examples, respectively.

Fig 5 illustrates the effects of autocorrelation on t-tests using synthetic data generated under the null hypothesis of no medication effect (as well as, of no time-of-the-day effect). To fix ideas let’s consider first the example with negative autocorrelation depicted in Fig 5a–5c. Here, we simulate 60 measurements of the outcome variable, *y*, using the model,

$$\begin{array}{c} y_{i}\;=\;\mu \;+\;\epsilon_{i}\;,\;\epsilon_{i}\;∼\;\text{AR}\left(\rho \right)\;,\;i=1,2,\ldots ,60\;, \end{array}$$ (7)

where *μ* represents an overall mean (note that the model does not contain treatment or time-of-the-day effect terms), and *ϵ*_*i*_ represents the residual error terms, simulated according to an autoregressive process of order 1 [*ϵ*_*i*_ = *ρϵ*_*i*−1_ + *γ*_*i*_, *γ*_*i*_ ∼ *N*(0, 1)], with an autocorrelation coefficient given by *ρ* = −0.95. The autocorrelation plot in Fig 5a shows strong negative autocorrelation for odd numbered lags and positive autocorrelation for even numbered lags, consistent with data generated from an autoregressive process with negative autocorrelation coefficient (since the value of the variable at time *t* is negatively associated with the value at time *t* + 1). Fig 5b shows the time-series of the outcome variable in the “paired” case, where a participant performs 2 activity tests per day, over a period of 30 days. The red and blue dots represent activities performed before and after the participant has taken medication, respectively. Note how the sequence of activities is perfectly regular across the 30 days, following the pattern “before”, “after”, “before”, “after”, …, “before”, “after”. Clearly, the data was simulated under the null hypothesis since the model used to generate the outcome variable (Eq 7) does not contain a medication or time-of-the-day effect term. Fig 5c shows the exact same outcome time-series in the “random” case, where a participant performs a single activity per day, over a period of 60 days, according to a random sequence of activities (“after”, “after”, “after”, “before”, …, “after”, “before”). Note that only the order of red and blue dots is different in panels b and c, but the outcome variable values, per se, are the same. However, the results from the t-tests are dramatically different in the “paired” and “random” cases (p-values equal to 1.67×10^−8^ and 0.99, respectively). In the paired case, the negative autocorrelation in the residuals leads to a pronounced separation of the red and blue dots since the negative association between outcome values at consecutive time points makes the time-series zig-zag around the mean (*μ* = 10, in this example), so that high or low outcome values tend to get synchronized with “before” or “after” activities. The random case, on the other hand, does not allow this synchronization, and we do not see a clear separation between the red and blue dots.

Fig 5d–5f illustrate the positive autocorrelation case, where we adopted *ρ* = 0.95 in the generation of the residuals. The autocorrelation plot in Fig 5d shows strong autocorrelations at both odd and even lags (up to lags 5 and 6), consistent with data generated from an autoregressive process with positive autocorrelation coefficient (since the value of the outcome variable at time *t* tends to be positively associated with the value at time *t* + 1). As a consequence, the outcome values at consecutive time points tend to be close to each other, and the time series tends to drift, rather than zig-zag around the mean. Again, the t-tests tend to show different behaviors in the “paired” and “random” cases (p-values equal to 0.94 and 0.85, respectively). In the paired case (Fig 5e), the strong association between the outcome values in consecutive time points means that every outcome value from a “before” activity (*i* = 1, 3, 5, …, 59) will be close to the consecutive outcome value in the “after” activity (*i* = 2, 4, 6, …, 60). Consequently, the average outcome values in the “before” and “after” populations tend to be very similar, and the t-test p-values tend to be larger than what we would expect by chance. Finally, Fig 8g–8i illustrate the case, where the residuals are independent (*ρ* = 0). Here, we see that the outcome time-series represents a middle ground between the negative and positive autocorrelation cases, showing more drift but less zig-zag than in the negative autocorrelation case, but less drift and more zig-zag than the positive correlation case.

To further illustrate the above points, we replicate the above 3 examples 10,000 times, and report the distributions of the estimated sample autocorrelations (lag = 1), and of the t-test p-values for the “paired” and “random” cases. Fig 5j–5l, show the distributions for the negative autocorrelation case. Note how the t-test tends to be anti-conservative (p-values smaller than they should be) in the “paired” case (Fig 5k), but exact (i.e., p-values follow a uniform distribution under the null) in the “random” case (Fig 5l). Fig 5m–5o, show the distributions for the positive autocorrelation case. Now, the t-test tends to be conservative (p-values larger than they should be) in the “paired” case (Fig 5n), but exact in the “random” case (Fig 5o). Finally, Fig 5p–5r, show the distributions for the case with no autocorrelation (*ρ* = 0). As expected, the p-value distributions are uniform for both the paired and random cases.

**Fig 6:**
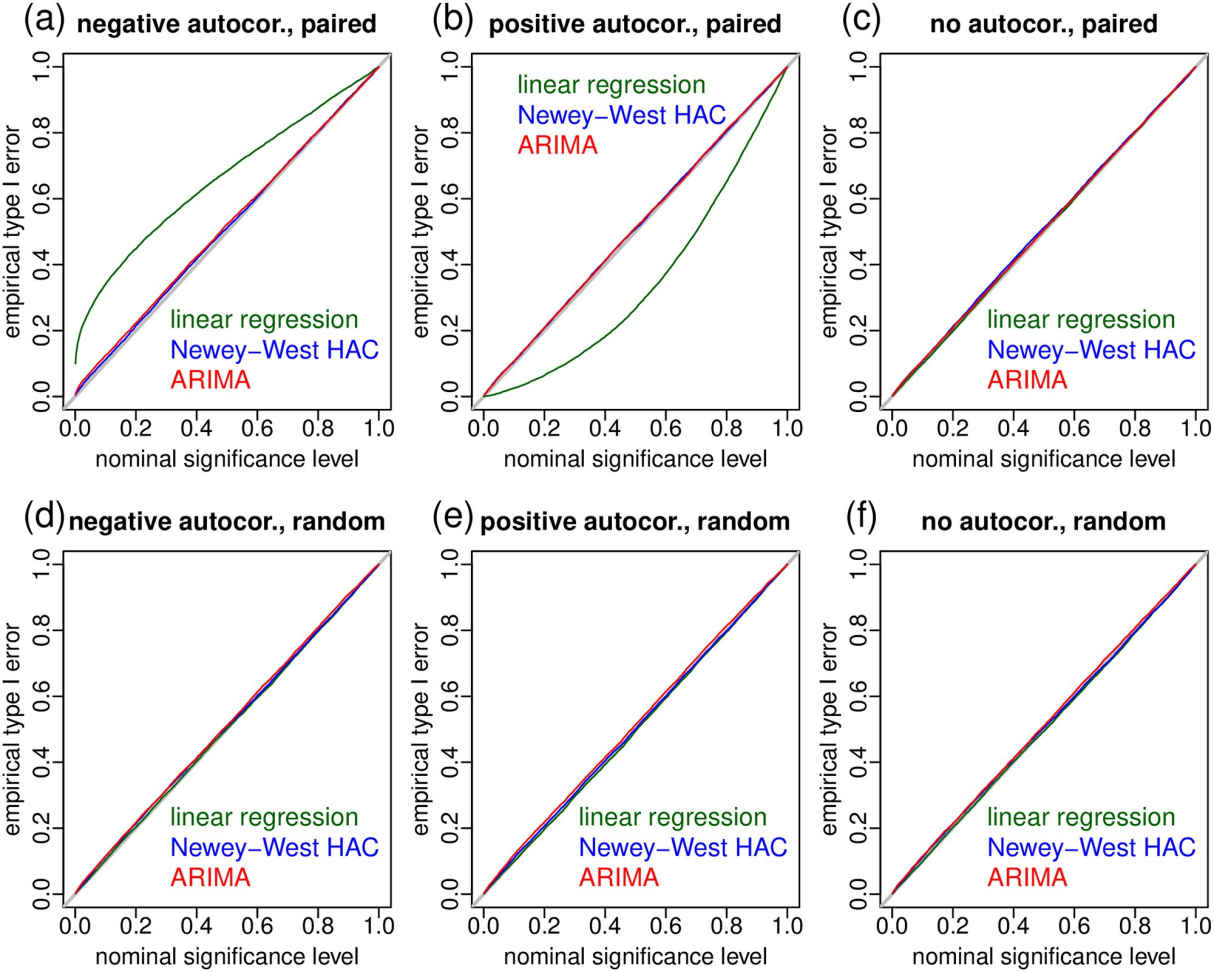
Assessing empirical type I error rates for the linear regression, Newey-West HAC, and ARIMA errors approaches. We run 3 separate simulation experiments for the negative (panels a and d), positive (panels b and e) and no autocorrelation (panels c and f) cases. Each experiment was based on 10,000 simulated data-sets generated according to the simulation parameters presented in Table 2. All panels report the nominal significance level (*α*) in the x-axis, and the respective empirical type I error rate in the y-axis (computed as the proportion of p-values smaller than the nominal significance level).

So far, we have illustrated in Fig 5 how serial autocorrelation can adversely impact the results of t-tests in the paired case (but not in the random case). Fig 6, on the other hand, illustrates how the Newey-West and ARIMA error regression approaches can handle residual autocorrelation even in the paired case. The figure reports empirical type I error rates from 6 simulation studies where we generated data under the null of no treatment effect (using the model in Eq 7) over a wide range of sample sizes and positive and negative autocorrelation strengths (described in Table 2), in both the paired and random cases. Each of the simulation experiments was based on 10,000 replications, and the empirical type I error rate was computed as the proportion of times that we rejected the null hypothesis across the 10,000 simulation replications. (Recall that a type I error corresponds to rejecting the null hypothesis when it is actually true. Since in our experiments the data was simulated under the null, we commit a type I error whenever we reject the null.) Each of the panels in Fig 6 report the empirical error rate in the y-axis against the nominal significance level (*α*) on the x-axis. The experiments show that the Newey-West (blue curves) and ARIMA (red curves) approaches are able to control the type I error rates at the nominal levels, since the empirical type I error rates closely track the nominal significance levels (i.e., at a nominal significance level of 0.05 the null hypothesis is rejected in approximately 5% of the simulations, at a significance level of 0.10 the null is rejected in approximately 10% of the simulations, and etc). This is true even in the paired case (Fig 6a – 6c). On the other hand, for the standard linear regression approach (green curves) we see in Fig 6a that the empirical type I errors tend to be higher than the nominal significance levels when the data shows negative autocorrelations (since the p-values tend to be smaller than they should), whereas in Fig 6b the empirical type I errors tend to be lower than the significance levels in the positive autocorrelation case (since the p-values tend to be larger than they should).

**Table 2:** Simulation parameter ranges for the simulation experiments reported in Fig 6. For each simulated data-set in each of the three experiments, we generated labels for both the paired and random cases, using a regular sequence of “before” and “after” labels in the paired case, and a random sequence in the random case (where, for each one of the *n* positions in the sequence, we randomly sampled a “before” or “after” label). Each data-set was generated with a distinct autocorrelation coefficient (*ρ*) value randomly sampled from the ranges described in the third column (autocorrelation) of the table, and with a distinct sample size (*n*) value randomly sampled within the range {30, 32, 34, …, 800}. (Note we always sampled even values of *n* to make sure we have complete “before” and “after” pairs in the paired case).

experiments	sample size (*n*) range	autocorrelation (*ρ*) range
experiment 1 (negative autocorrelation)	{30, 32, 34, …, 800}	[−0.9, 0]
experiment 2 (positive autocorrelation)	{30, 32, 34, …, 800}	[0, 0.9]
experiment 3 (no autocorrelation)	{30, 32, 34, …, 800}	0

### 2.4 Union-intersection tests for putative treatment effects and putative “time-of-the-day” effects

In Section 2.2, we described how to test for putative treatment and time-of-the-day effects for a single feature (outcome variable). In practice, however, we have multiple features and need to combine them into a single decision procedure. Here, we describe union-intersection (UI) tests for combining the feature specific tests into a single testing procedure.

Explicitly, suppose we have *p* features indexed from *k* = 1, …, *p*. The UI-test for a putative treatment effect is constructed by combining the feature specific tests,

$$\begin{array}{c} H_{0k}:\beta_{Y,X|T}=0\;\text{vs}\;H_{1k}:\beta_{Y,X|T}\neq 0\;, \end{array}$$ (8)

or,

$$\begin{array}{c} H_{0k}:\beta_{Y,X}=0\;\text{vs}\;H_{1k}:\beta_{Y,X}\neq 0\;, \end{array}$$ (9)

into a single test,

$$\begin{array}{c} H_{0}:\;\cap_{k=1}^{p}H_{0k}\;\text{vs}\;H_{1}:\;\cup_{k=1}^{p}H_{1k}\;, \end{array}$$ (10)

where we use the “time-of-the-day adjusted” test in (8) when the data associated with feature *k*, {*X*, *T*, *Y*_*k*_}, is consistent with the models *M*_2_, *M*_3_, *M*_5_, *M*_6_, *M*_8_, and *M*_9_ (for which, *T* → *Y*), and the “un-adjusted” test in (9) for models *M*_1_, *M*_4_, and *M*_7_ (for which, *T* is not a parent of *Y*). Note that because we are interested in detecting the (putative) direct causal effect of the treatment on the outcome, the choice to adjust or not for the time-of-the-day variable is tailored to the DAG structure (since the direct causal effect of *X* on *Y*, implied by a DAG, corresponds to the effect of *X* on *Y* conditional on all parents of *Y*, other than *X*).

Similarly, the UI-test for a putative time-of-the-day effect is built by combining the feature specific tests,

$$\begin{array}{c} H_{0k}:\beta_{Y,T|X}=0\;\text{vs}\;H_{1k}:\beta_{Y,T|X}\neq 0\;, \end{array}$$ (11)

or,

$$\begin{array}{c} H_{0k}:\beta_{Y,T}=0\;\text{vs}\;H_{1k}:\beta_{Y,T}\neq 0\;, \end{array}$$ (12)

into the single test where we use the treatment adjusted test in (11) when the data is consistent with models *M*_1_, *M*_3_, *M*_4_, *M*_6_, *M*_7_, and *M*_9_, and the un-adjusted test in (12) otherwise.

Described in words, the UI-test for putative treatment effect compares the null hypothesis of no putative treatment effect for all features, against the alternative that there is a putative treatment effect for at least one of the features. (Similarly, the UI-test for putative time-of-the-day effect compares the null hypothesis of no putative time-of-the-day effect for all features, against the alternative that there is a putative time-of-the-day effect for at least one of the features.) Under this test, we reject the null if the p-value of at least one of the feature-specific tests is small. Hence, the p-value for the UI-test corresponds to the smallest p-value (across all *p* features) after multiple testing correction. The UI-tests can be constructed using the output of any of the 3 linear regression approaches (standard, ARIMA errors, and Newey-West) described in the previous section. Note, as well, that when this personalized UI-test is (separately) applied to multiple participants, is is necessary to perform a second round of multiple testing correction across the participant’s UI-test p-values.

## 3 Real data illustrations

We illustrate the methodology proposed in this paper using data collected by the mPower study [11], a mobile health study in Parkinson’s disease (PD) approved by Western Institutional Review Board (WIRB protocol #20141369), and registered at ClinicalTrials.gov (identifier #NCT02696603). The study was open to individuals with and without PD, and informed consent was obtained via an interactive, in-app eConsent process that included a quiz on the risks, benefits, and options for study participation and data sharing. Enrollment required participants to answer all questions correctly (although participants could take the quiz multiple times) [27].

We investigated whether participants that self-reported as PD patients showed a response to dopaminergic medication by comparing their performance in tapping activity tasks performed before the participant has taken medication versus after medication. PD patients are usually treated with medications that reduce disease symptoms (with dopaminergic medications representing the standard treatment). While treatment effectively reduces symptoms in some patients, others do not respond well to medication and experience fluctuations in symptom severity throughout the day [28]. Because some participants in the mPower study tended to take their medication at the same time every day, the evaluation of medication effects might be confounded with diurnal factors associated with the time-of-the-day that the activity was performed. Hence, it is important to evaluated whether variation in performance in the tapping activity reflects (putative) medication effects, temporal effects or still both medication and temporal effects concomitantly.

For the tapping activity, participants were instructed to lay their smartphone on a flat surface and to use two fingers of the same hand to alternatively tap two stationary points on the phone screen for 20 seconds. We focused the analyses on 99 PD patients that performed at least 15 tapping activities before taking medication and 15 activities after medication and that consented to share their data with qualified researchers for secondary analyses. The number of activities per participant ranged from 31 to 445, with 1st-quartile, median, mean, and 3rd-quartile given, respectively, by 60.5, 91, 120.6, and 142.5. (Note that reference [3] presents analogous analyses based on a larger set of subjects including participants that did not consent to use their data for secondary analyses).

The longitudinal data of each participant was analyzed separately. The analyses were based on 41 features extracted from the raw tapping data collected by each activity. (For each activity, the raw tapping data corresponds to a time series of screen pixel positions of where participants tapped the screen, together with the time-stamps of the touches.) Extracted features included the total number of taps, summary statistics on the tapping intervals between two points, summary statistics on the drift from each point, among others (see reference [29] for a description). The data from each extracted feature was separately de-trended with a lowess smoother, so that our feature data actually corresponds to the residuals of a lowess fit to the data point collection index. (De-trending the data is necessary to avoid learning trend artifacts, where a participant’s performance in an activity task gets better over time as the participant gets more used to it. This can be an artifact in situations where, for example, a participant tends to perform activities before medication at a higher frequency in the beginning of the study, before switching to performing after medication activities at a higher frequency later on, and vice-versa.) The data was also transformed to an approximately normal distribution using a rank-quantile transformation, Φ^−1^((*r*_*i*_−0.5)/*n*), where Φ() represents the cumulative density function of the standard normal random variable, *r*_*i*_ represents the rank of the outcome value, *y*_*i*_, and *n* represents the number of outcome data points. Additionally, because time-of-the-day is a circular variable, we have that the linear term used in our models for encoding this variable treats values such as 23:59 and 00:01 very differently (even though these values are only 2 minutes apart). To avoid potential issues arising from the circularity of the time-of-day-variable, we filtered out any activities (records) that were performed between midnight and 5am.

**Fig 7:**
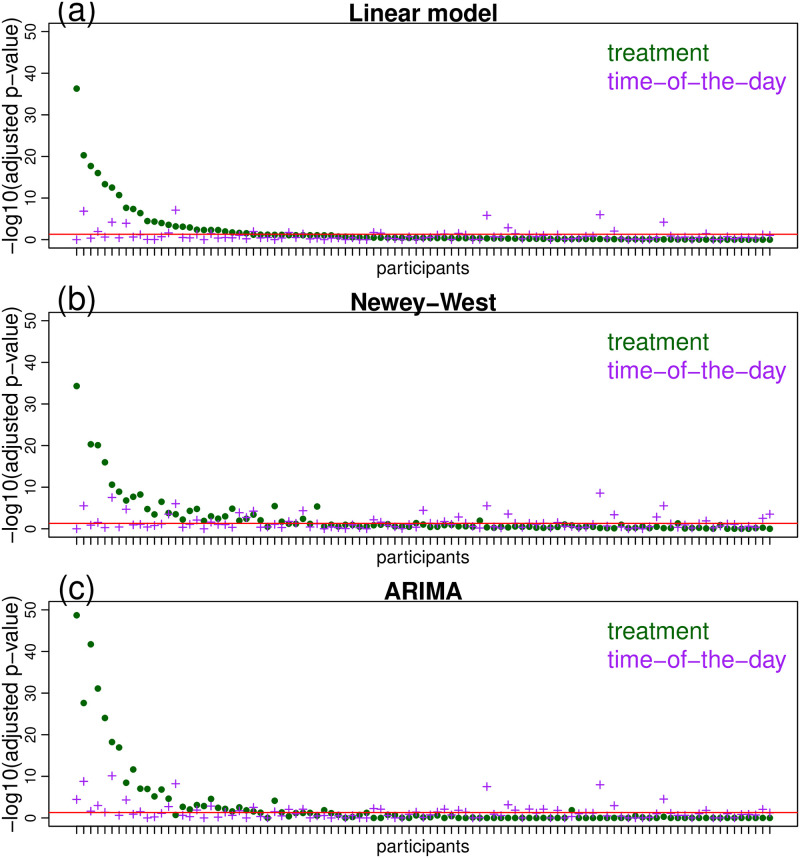
Personalized response to putative medication and time-of-the-day effects. Panels a, b and c show, respectively, the adjusted p-values (in -log_10_ scale) from the union-intersection tests for putative medication effects (green dots) and putative time-of-the-day effects (purple plus signs), for the linear regression, Newey-West HAC covariance estimation, and ARIMA error models. The red horizontal lines correspond to a p-value threshold of 0.05. The order of the participants in the x-axis is the same for all panels, with the participants sorted according to the putative treatment p-value from the linear regression model (green dots) in panel a.

Before applying the time series techniques, we first analyzed the data using the standard linear regression approach (as a naive baseline method which ignores the time series structure of the data). Fig 7a report the results and suggests that approximately 18%, 14%, and 7% of the participants showed putative medication responses, putative time-of-the-day effects, or still both medication and time-of-the-day responses, according to our union-intersection tests after Benjamini-Hochberg multiple testing correction at 5% FDR across the participants (we also used Benjamini-Hochberg correction across the 41 tapping features, when computing the UI-test p-values). However, as described before, whether residual autocorrelation impacts the type I error rates of the t-tests underlying our union-intersection tests, depends on whether a participant performs the activity tasks in a paired or unpaired (and close to random) fashion.

**Fig 8:**
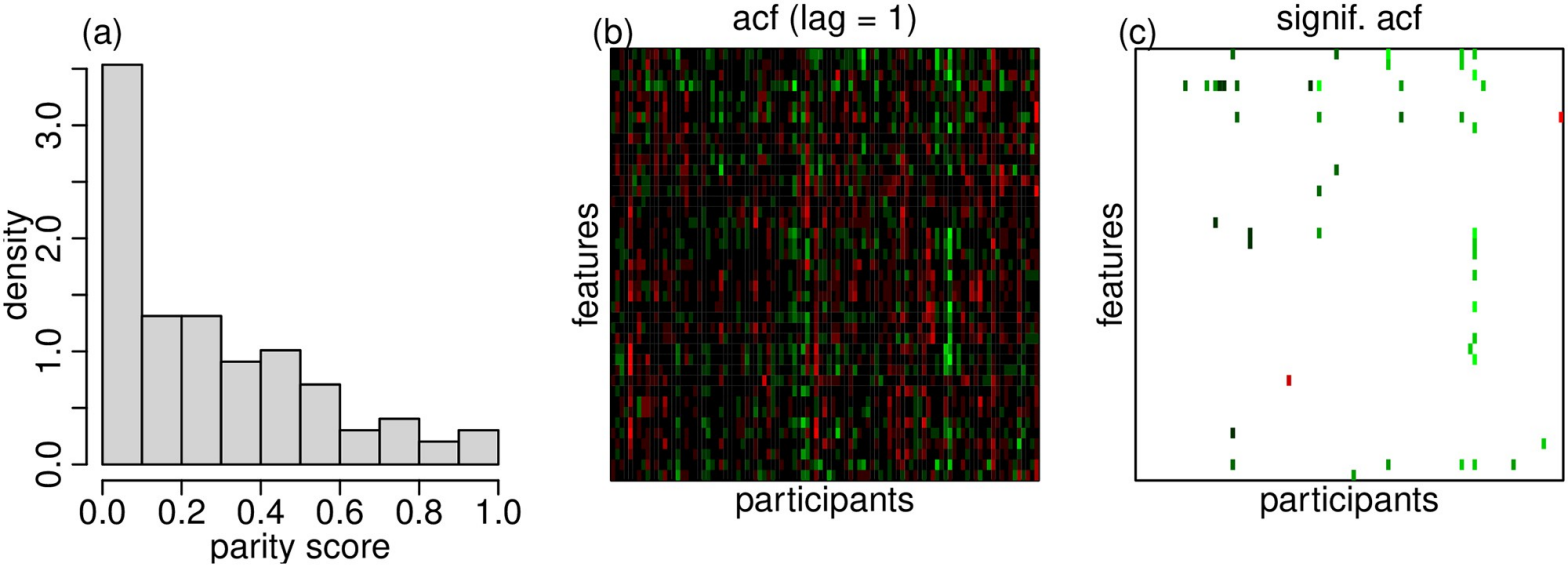
Panel a shows the distribution of the “parity score” across all participants. The parity score was defined as the proportion of days where the participant performed the tapping task before and after taking medication on the same day. Panel b shows a heatmap of the residual autocorrelation (lag = 1) of the linear regression model fits across all feature/participant combinations. Red and green represents, respectively, negative and positive autocorrelation. Panel c shows only the autocorrelation values that were statistically different from zero according to multiple testing corrected Ljung-Box tests at a significance threshold of 0.05. (The autocorrelation values that were not statistically significant are shown in white.) Only about 4.1% of the statistically significant autocorrelations were negative.

In the mPower study, it is the participant who decides whether he/she will perform the activity before or after taking medication. Inspection of the mPower’s before/after label data shows that while a larger fraction of participants seen to have performed the activities closer to the unpaired pattern, there is still a certain number of participants that tended to perform the activities closer to a paired fashion (Fig 8a). This suggests the need for time series techniques.

Re-analyses of the data using regression with Newey-West HAC covariance estimation and regression with ARIMA errors shows that modeling the autocorrelation structure tended to increase the number of significant putative effects in most cases, as described in Table 3. Fig 7b and 7c present the results of our union-intersection tests based on the Newey-West HAC and ARIMA approaches, respectively. (Similarly to the synthetic data experiments, we adopted the Newey-West HAC estimator, using Bartlett kernel, and the automatic bandwidth selection procedure implemented in the sandwich R package [25], and ARIMA residual modeling using the auto.arima function of the forecast R package [23] in order to first select the autoregressive, moving average, and differencing orders of the models that are used for the hypothesis testing).

**Table 3:** Proportion of participants showing statistically significant putative effects, across the 3 distinct analysis approaches (after multiple testing correction). The abbreviation t.o.d. stands for time-of-the-day. The average column represents the average across the three methods.

Putative effect	Linear regression	Newey-West	ARIMA	average
medication alone	18%	21%	14%	18%
t.o.d. alone	14%	22%	21%	19%
both	7%	12%	15%	11%

The larger number of significant results obtained by the Newey-West and ARIMA regressions suggest that the naive linear model might have been slightly conservative in this data set, perhaps due to positive autocorrelations in the data. (Recall that, as described in Section 2.3, positive autocorrelations tend to produce conservative results for the t-tests performed by the standard regression model.) Inspection of the autocorrelation in the residuals of the naive regression model fits shows that, as expected, most of the statistically significant autocorrelations were indeed positive (Fig 8b and 8c).

Observe that apart from the overall higher number of significant putative effects, Fig 7 illustrates that the results from the naive regression approach are still largely consistent with the time series approaches. This is not surprising given that most participants performed the activities in a way that was closer to an unpaired pattern (Fig 8a), where time series methods are not really needed.

Overall, when averaging the results across all three approaches, our analyses suggest that: (i) approximately 52% of the participants did not show medication or time-of-the-day effects; (ii) approximately 29% of the participants showed effects that could possibly be attributed to medication (with 18% showing putative medication effects alone); (iii) approximately 30% of the participants showed effects that could be attributed to time-of-the-day (with 19% attributed to time-of-the-day alone); and (iv) the concomitant presence of medication and time-of-the-day effects (11%) tended to be less common than either of these effects alone.

## 4 Discussion

In this work we proposed a statistical approach to tell apart putative treatment effects from putative “time-of-the-day” effects in observational studies. The ability to disentangle these two effects is important in practice, since any causal inferences about personalized treatment effects are especially vulnerable to daily cyclic confounding factors, such as circadian rhythms and daily routine activities.

The key insight that makes the approach practical is the realization that every time that an activity is performed, the measurement of the treatment and time-of-the-day variables precedes the measurement of the outcome variable, so that any causal models where the outcome plays the role of a cause of the treatment or time-of-the-day variables are automatically disregarded. This allows the use of just a few conditional independence relationships to distinguish between putative treatment and time-of-the-day effects, irrespective of the causal relation between treatment and time-of-the-day variables.

Another contribution of the paper, is to clarify the conditions under which autocorrelation in the measured outcomes can invalidate conditional independence tests in linear models. In particular, we illustrate how serial autocorrelation can adversely impact the results of standard t-tests in situations where the participants tend to perform the activities in a paired fashion, but not when the participants tend to perform the before medication and after medication activities in an irregular order. While no time series techniques are needed in the latter case, we adopt temporal regression models as remedies for autocorrelation issues in the former case. Still another contribution of the paper, is the use of union-intersection tests to aggregate evidence across multiple outcomes (features) into a single statistical test. UI-tests have been used before in mobile health studies in a simpler setting considering only treatment effects [7]. Here, we extent it to the context of both treatment and time-of-the-day effects.

In the present study we investigated the performance of regression with ARIMA errors and robust regression with HAC covariance estimation (based on Newey-West estimator). One caveat of these approaches is that they assume that the data is equally spaced, what is not true in our application. It has been shown, nonetheless, that application of the Newey-West estimator to time series with unequally spaced data still generates asymptotically consistent estimates of the covariance matrix, as well as, reasonable performance in finite sample simulation studies [30, 31].

There is a vast literature on causal inference for time series data (see [32] for a recent review). In the particular context of causal discovery (where the goal is to identify causal relationships between distinct time series) the main approaches can be classified into Granger causality, traditional causal discovery approaches adapted to time series, and deep learning based methods.

Granger causality approaches [33, 34] are based in the idea that a time series *X* is said to Granger cause a time series *Y* if the prediction of the time series *Y* is improved by allowing lagged values of the *X* and *Y* time series to improve the prediction of future values of *Y*. The approach is implemented using linear models usually through a series of t- or F-tests on the lagged values of *X* and *Y*, and its main advantage is its computational simplicity. In its original form, Granger causality does not capture contemporaneous and non-linear causal relationships, nor does it accounts for latent confounding. It has, nonetheless, been extended in several directions including for vector autoregressive models [35, 36], non-linear additive models [37], and partial Granger causality approaches [38, 39] which can deal with exogenous and latent variables.

As pointed by [40], traditional causal discovery algorithms can be classified into: (i) constraint-based methods which use conditional independence tests to find causal skeletons and determine orientations up to the Markov equivalence class (widely-used methods include PC and FCI [14] algorithms); (ii) score-based methods which adopt a scoring function that measures how well an equivalence class fits the observed data and search through equivalence classes to find the best scored one [41–43], and (iii) functional causal model-based approaches which exploit asymmetries between causal and anti-causal directions by assuming certain constraints on the class of causal mechanisms [44–46]. All these approaches have been successfully adapted for the analysis of time series data.

In the context of linear systems with joint normal distributions, and under the assumption of no unmeasured confounding, the approaches proposed by [47–49] adapt the PC algorithm [14] for performing causal discovery in vector autoregressive models. In order to allow for latent confounding other approaches have adapted the FCI algorithm [14] to causal discovery in time-series [50, 51]. Additionally, several other constraint- and score-based approaches aiming to handle non-linear time-series have been proposed in the literature [51–60].

Functional causal models approaches, based on the linear non-gaussian acyclic model (LINGAM) proposed by [44], have also been adapted for causal discovery in time series data. For instance, [61] proposed the time series LINGAM model which allows for contemporaneous effects, but not for confounding, while [62] extended the LINGAM model to learn linear cyclic models in the presence of latent confounders, and [63] integrated LINGAM with tensor based techniques for performing causal discovery in high dimensional data. Additionally, [64] has proposed a functional causal model approach that leverages non-stationarity for aiding causal discovery.

While this rich literature include methodologies for dealing with non-linearity, non-gaussianity, the presence of unmeasured confounding, most of these methods do not allow for contemporaneous causal relations (i.e., causal effects between variables at the same time point). Exceptions include the works of [51, 53, 54, 64] which can handle both contemporaneous and dynamic (lagged) causal relations.

Deep learning based approaches have also been recently proposed for performing causal discovery in time series data [65–72]. These highly flexible models are able to detect non-linear and time-variant relations [66], model non-stationarity [72], account for unobserved confounders [69], and can even be used to infer causal relations across samples with different underlying causal graphs but shared dynamics [71]. The main disadvantage of deep learning based approaches is that they usually require large sample sizes compared to the simpler approaches.

Outside the context of causal discovery, there is also a rich literature in causal treatment effect estimation for time series data. This literature can be classified in two main areas: (i) estimation of time-invariant effects, where the causal effect is assumed to be constant over time; and (ii) the estimation of time-varying treatment effects, where the causal effects are allowed to change through time. See [32] and references within for further details on these treatment effect estimation methods.

As pointed out before, our approach is closer in spirit to constraint-based causal discovery algorithms. (Recall that the temporal regression model fits described in Eqs (2) to (5) are just a convenient way to perform conditional independence tests in temporal regression models. Observe, as well, that we are not really interested in estimating causal effects. The actual goal is to select the causal graph, among the 9 causal models in Fig 2, based on the observed conditional independencies in the data).

One important distinction of our proposed methodology relative to the other constraint-based approaches in the literature (which focus mainly on dynamic effects, i.e., lagged effects) is that we are only interested in detecting contemporaneous effects. (Note that in our application we are interested in modeling the effects of a fast-acting medication and/or of the time-of-the-day on a participant’s performance in a tapping activity task. We are only interested in contemporaneous effects since taking a fast acting medication today should not have an influence on a participant’s symptoms tomorrow, and, similarly, we should not expect that the time-of-the-day that a participant performed an activity today should influence the participant’s performance tomorrow. Only contemporaneous effects at the same time point play a role in our application).

Another distinction of our approach is that it does not require the explicit specification of the serial association structure of our data. Note that while the other few constraint-based methods that allow for contemporaneous effects in the literature require the specification of a time series model (e.g., the method proposed by [53] requires the user to specify the lag of the scatterplot smoothers in the additive non-linear time-series model), our proposed approach, on the other hand, either learns the serial association structure of the regression residuals automatically from the data based on the ARIMA model selection procedure described in reference [23], or adjusts for autocorrelation and heteroscedasticity (of unknown form) using robust regression based of HAC covariance estimation. This is an important practical advantage in applications (such as ours) where we need to analyse dozens of time series models. (Recall that we fit temporal regression models to multiple sensor based features. In our Parkinson’s disease illustration we analyzed 41 distinct time series of features extracted from the tapping activity, but in other applications this number can be considerably larger.). Another important distinction of our work relative to the current literature is that it combines the analyses of the multiple time series into a single decision procedure based on union-intersection tests.

Our approach, however, has a few important limitations. First, it assumes linear relationships, and that the causal effects between these variables are constant over time. Second, while longer-term confounding artifacts are not very likely, it is, nonetheless, still possible that our results might be biased to some extent by these sources of unmeasured confounding. Interesting research questions (which are nonetheless outside the scope of the present paper) include how to extend our approach to account (in a computationally efficient way) for non-linear associations, un-measured confounding, and applications where the strength of the causal relations might change over time.

Despite its limitations, the approach proposed in this paper represents a first step towards the problem of disentangling personalized medication effects from time-of-the-day effects in observational mobile health studies, and we believe that the mobile health community will find this tool useful for other applications assessing personalized treatments in observational studies.
